# Determination of Poisson Ratio of Bovine Extraocular Muscle by Computed X-Ray Tomography

**DOI:** 10.1155/2013/197479

**Published:** 2012-12-30

**Authors:** Hansang Kim, Lawrence Yoo, Andrew Shin, Joseph L. Demer

**Affiliations:** ^1^Department of Mechanical and Automotive Engineering, Gachon University, Seongnam-Si, Gyeonggi-do 461-701, Republic of Korea; ^2^Department of Ophthalmology, Jules Stein Eye Institute, University of California, Los Angeles, CA 90095-7002, USA; ^3^Department of Mechanical Engineering, University of California, Los Angeles, CA, USA; ^4^Biomedical Engineering Interdepartmental Program, University of California, Los Angeles, CA, USA; ^5^Neuroscience Interdepartmental Program, University of California, Los Angeles, CA, USA; ^6^Department of Neurology, University of California, Los Angeles, CA, USA

## Abstract

The Poisson ratio (PR) is a fundamental mechanical parameter that approximates the ratio of relative change in cross sectional area to tensile elongation. However, the PR of extraocular muscle (EOM) is almost never measured because of experimental constraints. The problem was overcome by determining changes in EOM dimensions using computed X-ray tomography (CT) at microscopic resolution during tensile elongation to determine transverse strain indicated by the change in cross-section. Fresh bovine EOM specimens were prepared. Specimens were clamped in a tensile fixture within a CT scanner (SkyScan, Belgium) with temperature and humidity control and stretched up to 35% of initial length. Sets of 500–800 contiguous CT images were obtained at 10-micron resolution before and after tensile loading. Digital 3D models were then built and discretized into 6–8-micron-thick elements. Changes in longitudinal thickness of each microscopic element were determined to calculate strain. Green's theorem was used to calculate areal strain in transverse directions orthogonal to the stretching direction. The mean PR from discretized 3D models for every microscopic element in 14 EOM specimens averaged 0.457 ± 0.004 (SD). The measured PR of bovine EOM is thus near the limit of incompressibility.

## 1. Introduction

Since extraocular muscles (EOMs) are manipulated mechanically during strabismus surgery to correct binocular misalignment, the mechanical properties of the EOMs should be understood in order to optimize surgical results. With increasing demand for accuracy in simulation of orbital mechanics, finite element analysis (FEA) is becoming increasingly attractive. However, casual estimation of mechanical parameters in FEA can lead to serious errors in simulation. In order to accurately determine the biomechanical properties of orbital tissues, scientists in the field have employed a variety of experimental techniques. While conventional tensile elongation tests have been performed to investigate the uniaxial force and length relationship for EOMs [1–5], micro/nano indentation has permitted measurement of the compressive modulus of other orbital tissues [6, 7]. Despite such efforts [1, 5–11], many material parameters of orbital tissues have yet to be defined. Experimental technique and the theoretical constitutive framework should be appropriate for each tested orbital tissue. For instance, triborheometry, which treats a solid from rheometric perspective, was employed for characterizing amorphous specimens such as orbital connective and fatty tissue [10]. A variety of other biomechanical methods [12] have been employed to characterize more comprehensive constitutive models for EOMs that capture their time-dependent relationships between stress and strain [1, 5, 8]. 

The Poisson ratio (PR) is a critical mechanical parameter required to define comprehensively the elastic behavior of a material. The PR is the ratio of the transverse contraction strain to the axial extension strain, which can be obtained during simple tensile elongation. Normally, the PR of a material ranges between 0 and 0.5, depending on the material's compressibility. However, the PR can be higher than 0.5 or lower than 0 for materials having complex matrices and inner structures [13, 14]. Most soft tissues are considered to be elastomeric materials with high bulk modulus relative to Young's modulus, so the PR is expected to approximate 0.5 [15]. 

In general, the PR can be measured by static or dynamic methods. Static methods, such as classical tensile or compressive testing, are most widely used in solid mechanics [15, 16]. In static determinations, the PR is calculated from transverse and axial deformations due to uniaxial stress. For dynamic determination, the PR is determined from the natural frequency of the transverse and axial waves in the material [17–19], most commonly elicited by ultrasound perturbation. 

The PR has been typically assumed to be between 0.35 and 0.49 for soft tissues [20–22]. However, it has been estimated that a 20% error in the PR would result in errors of 3.8% and 4.4% in the biaxial flexural strength and the indentation modulus of a material, respectively [15]. Such errors could propagate and compound during the iterative computations in FEA. Clearly, there is a need to minimize errors by accurate experimental determination of the PR. By employing a novel X-ray computed tomographic (CT) imaging method for precise determination of strain, we aimed to extract accurate static PR for EOMs.

## 2. Materials and Methods

### 2.1. Specimen Preparation

Fresh heads of adult cows were obtained from a nearby abattoir. In the laboratory, orbits were carefully dissected for extraction of EOM and connective tissue. Transport time from abattoir to laboratory was approximately 30 min; the additional time elapsed to dissect the EOMs averaged 3 hrs. After extraction, EOMs were maintained in lactated Ringer's solution at 37°C. To minimize axial damage to EOM fibers, each specimen was initially prepared in the shape of an approximately 7 mm long prism with a 4 mm × 2 mm cross-section. For consistency, samples were prepared from the transverse center of each EOM. Given that clamping of both ends was necessary, the actual tested length was the 10 mm of middle portion of each specimen and in every case avoided the terminal tendon. Specimen preparation time was approximately 45 min.

### 2.2. Experiment

**Figure 1:**
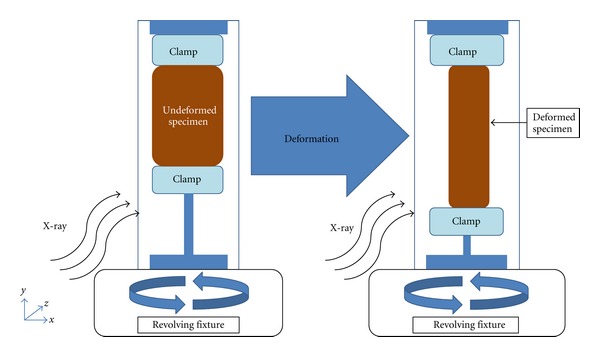
Both undeformed and deformed states of EOM specimens were imaged. As shown above, the X-ray direction was orthogonal to the revolving specimen fixture.

A high-resolution micro-CT scanner (Model 1172, SkyScan, Belgium) incorporating a tensile loading fixture was used to image deformed and undeformed states of 14 freshly prepared bovine EOM specimens. Scanning was accomplished by revolving and longitudinally translating the specimen and tensile fixture between a fixed, collimated X-ray source and a fixed detector (Figure 1). Once loaded in the tensile fixture, the undeformed specimen was first imaged at 10 micron spatial resolution. After the specimen was elongated 30%–35%, which is well within the linear elastic region [1], the deformed specimen was again imaged. In order to prevent dehydration, corn oil was applied on each specimen before placement into the tensile fixture. Figure 1 shows schematics of undeformed and deformed states of the specimen in the tensile fixture.

### 2.3. 3D Reconstruction of EOMs

After EOM specimens were scanned, 500 cross-sectional area (CSA) images for undeformed and 800 CSA images for deformed states were used to create 3D reconstruction of the entire length of each EOM specimen using Matlab (Version R2010a, The MathWorks, Inc., Massachusetts) image processing tools and SolidWorks CAD software (version 2011, Dassault Systèmes SolidWorks Corp., Massachusetts). More deformed than undeformed image planes were required since the deformed specimen was elongated. The CSA in each image plane was connected to that in the adjacent planes in order to generate 3D reconstruction by using the loft feature in Solidworks.

### 2.4. Poisson Ratio

As shown in (1), the PR *ν*, for materials undergoing deformations exceeding 1% [23], is expressed as the negative ratio of transverse to axial true strain [24, 25]:

(1) $$\begin{array}{c} \nu =-\frac{\ln\left(1+\varepsilon_{T}\right)}{\ln\left(1+\varepsilon_{A}\right)}, \end{array}$$

where *ε*_*T*_ and *ε*_*A*_ are the transverse and axial engineering strains, respectively. Equation (1) can be rearranged and can be expressed as shown in (2):

(2) $$\begin{array}{c} {\ln\left(1+\varepsilon_{A}\right)}^{-\nu }=\ln\left(1+\varepsilon_{T}\right). \end{array}$$

Recognizing the fact that an infinitesimal element in the CSA undergoes a plane deformation when the EOM is subjected to loading, the transverse strain in (2) can be shown as ln⁡(*δx*/*δx*_*ο*_) and (2) can be rearranged as (3) with *δx*_o_ and *δx* being the length of side of the element in undeformed and deformed configuration:

(3) $$\begin{array}{c} \delta x=\delta x_{ο}{\left(1+\varepsilon_{A}\right)}^{-\nu }. \end{array}$$

Equation (3) is valid under the assumption of isotropy or transverse isotropy, which is appropriate for an EOM [25]. Hence the CSA of the square in the deformed configuration can be expressed as (4) where *δA*_0_ is the initial CSA:

(4) $$\begin{array}{c} \delta A=\delta {x_{ο}}^{2}{\left(1+\varepsilon_{A}\right)}^{-2\nu }=\delta A_{ο}{\left(1+\varepsilon_{A}\right)}^{-2\nu }. \end{array}$$

As reported by Vergari et al. [25], the instantaneous CSA can be expressed as (5) by summation of all the elements in the EOM CSA:

(5) $$\begin{array}{c} A=A_{ο}{\left(1+\varepsilon_{A}\right)}^{-2\nu }, \end{array}$$

where *A* and *A*_*ο*_ are the instantaneous and initial EOM CSA values, respectively. The CT scanner employed was specifically designed to image CSAs suitable for 3D reconstruction. After 3D reconstruction for both deformed and undeformed states, each model was then uniformly discretized into 8 micron thick elements. The CSA for each element was then computed using Green's theorem as shown in (6):

(6) $$\begin{array}{c} A=\oint_{C}xdy=-\oint_{C}ydx=\frac{1}{2}\int_{C}\left(-ydx+xdy\right). \end{array}$$

Finally, the PR for each discretized element was calculated using (7):

(7) $$\begin{array}{c} \nu =-0.5\frac{\ln\left(A/A_{ο}\right)}{\ln\left(1+\varepsilon_{A}\right)}. \end{array}$$

For more precise evaluation of PR, the CAD surface reconstruction excluded regions near clamping plates that are influenced by the clamping forces. After the PR was computed for all the elements within each specimen using (7), the average PR was calculated for each of the 14 specimens tested. 

## 3. Results 

### 3.1. 3D Reconstruction

**Figure 2:**
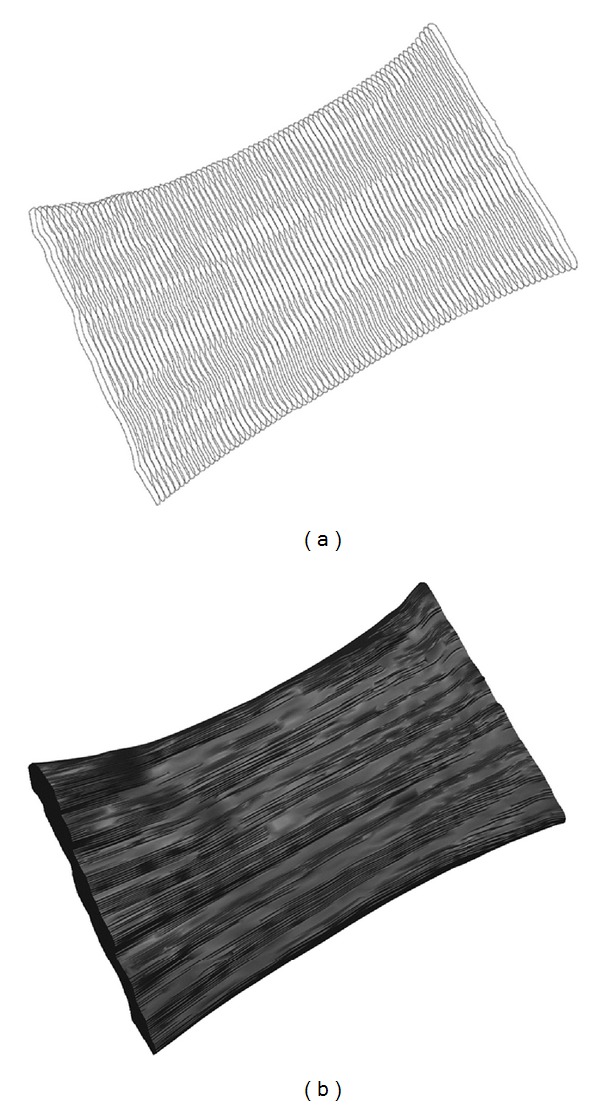
(a) Decimated set of CSA images for an undeformed EOM specimen. (b) Finished 3D reconstruction from 500 CSAs.

Figure 2 shows the 3D reconstruction from 500 CSAs (Figure 2(a)) used to build the undeformed model of an EOM specimen (Figure 2(b)). 

**Figure 3:**
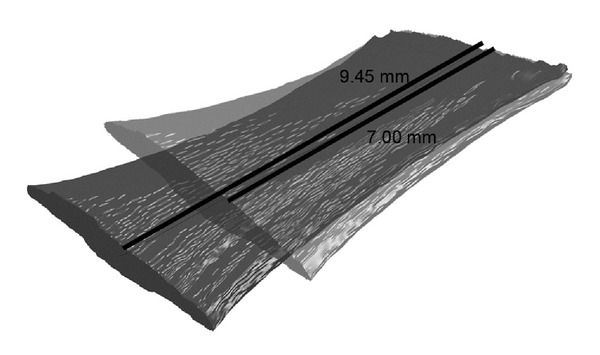
Finished 3D models for both undeformed and deformed states of an EOM specimen. The original length of the specimen was 7 mm, and the final length of the specimen after the 35% deformation was 9.45 mm.

Once 3D reconstruction was completed for each specimen for both undeformed and deformed states, the models were discretized into elements with uniform thickness of 6 microns and 8.1 microns, respectively. During preliminary experiments it was verified that all 6 anatomical EOMs exhibited similar PR values. Thus PR values for 6 EOMs were not differentiated by anatomical EOM.  Figure 3 contrasts undeformed and deformed states of the same EOM specimen.

**Figure 4:**
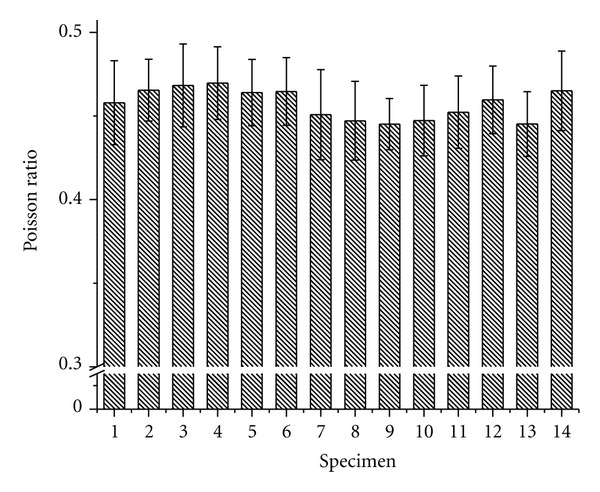
PR values for all 14 specimens. Error bars indicate SD.

Assuming uniform stretch throughout each specimen, the PR for each element within each EOM specimen was computed from the CSAs and actual elongated length.  Figure 4 shows the average PR values for each of the 14 specimens.

The PR over all 14 specimens averaged 0.457 ± 0.004 (standard deviation (SD)) was highly significantly different by Student's *t*-test from the ideal incompressible value 0.5 (*P* < 10^−9^).

## 4. Discussion

Micro-CT imaging of EOM specimens effectively characterized the PR for bovine EOM during tensile elongation, which is a critical mechanical parameter. The present investigation is the first to evaluate bovine EOM PR directly from CSA measurements by noncontact imaging during large deformation. Prior studies were based on measurement of small, linear transverse deformations [26, 27]. In the present investigation, mean PR of 14 bovine EOM was computed to be 0.457 ± 0.004 (SD). While isotropic media cannot have PRs exceeding 0.5, orthotropic or transversely isotropic materials (such as tendons) sometimes have PRs exceeding 1 [25]. A PR value approximating 0.5 has interesting implications for tendon behavior: assuming conservation of mass, a PR value smaller than 0.5 implies an increase in volume during loading leading to a decrease in density. On the other hand, a PR exceeding 0.5 implies a volume reduction and an increase in density. Still, the mean PR for all EOM specimens tested in the present study was 0.457, so EOM under axial loading can be considered to a good approximation to behave as an incompressible material. The small volume variations measured might be caused by water loss, as suggested by Lynch et al. [27]. However, since in the present experiment corn oil was employed to coat the specimen to avoid dehydration, measured small volume variations are probably due to internal rearrangements of the fiber structures [25]. The precise PR value for EOM reported in the present study should facilitate quantitative modeling of ocular motor biomechanics and is represented in a theoretical framework practical for graphical simulation of quasistatic ocular motility using FEM. 

 The present paper introduces micro-CT imaging as a noncontacting approach to compute the PR from specimen geometry. As it has been presented, micro-CT imaging technology coupled with 3D reconstruction based on the volumetric specimen changes allows more accurate evaluation of Poisson's ratio for bovine EOM specimens. 

## 5. Conclusion 

The current investigation describes a method to determine the PR for bovine EOM based upon CSAs from discretized deformed and undeformed specimens obtained from micro-CT imaging during quasistatic loading. The study also demonstrated that 3D reconstruction of micro CT imaging can be successfully performed for both deformed and undeformed EOM specimens. In the present study, the PR, a critical parameter for quantitative mechanical characterization of soft tissues that is necessary for modeling and simulation, was determined for bovine EOM specimens to be near the criterion for incompressibility.
